# Einstellung gegenüber Online-Beratung: Eine Umfrage unter Berater:innen, Coaches und Therapeut:innen

**DOI:** 10.1365/s40896-021-00061-5

**Published:** 2021-12-22

**Authors:** Cindy Römer, Lukas Mundelsee

**Affiliations:** Erziehungswissenschaftliche Fakultät, Universität Erfurt, Nordhäuser Straße 63, 99089 Erfurt, Deutschland

**Keywords:** Einstellungen, Online-Beratung, Online-Therapie, Online-Coaching, Prädiktoren, Attitudes, Online counselling, Online therapy, Online coaching, Predictors

## Abstract

Spätestens seit der Corona-Pandemie ist das Thema „Digitalisierung“ auch in der Beratungswelt angekommen. Online-Beratung wird von vielen Praktizierenden jedoch mit großer Skepsis begegnet. Was genau diese negative Einstellung verursacht, wurde bislang nur wenig untersucht. Bisherige Studien bezogen sich entweder auf Video-Therapien oder auf die Einführung evidenzbasierter Praktiken. Ziel der vorliegenden Querschnittsstudie war es deshalb, mögliche Merkmale zu ermitteln, die die Einstellung gegenüber Online-Beratung im Vergleich zur Präsenz-Beratung positiv oder negativ beeinflussen. Zur Erfassung der Einstellung wurde ein neues Instrument entwickelt, das verschiedene Merkmale von Beratung beschreibt, und mittels Online-Fragebogen bei 66 Personen aus den Bereichen Beratung, Therapie und Coaching eingesetzt. Faktorenanalytisch ergaben sich bei dem Instrument zwei unterschiedliche Einstellungsdimensionen, die in den anschließenden Regressionsanalysen von jeweils unterschiedlichen Personenmerkmalen prädiziert wurden. In Bezug auf die Dimension „Kernmerkmale von Beratungen“ wird eine negativere Einstellung nur durch eine höhere Berufserfahrung vorhergesagt. Hinsichtlich der Dimension „Rahmenbedingungen von Beratungen“ zeigt sich eine positivere Einstellung gegenüber dem Online-Setting, wenn die Beratenden sich als Frau identifizieren, eine geringere Berufserfahrung vorweisen, im Bereich Coaching tätig sind oder an urbanen Orten praktizieren. Interessanterweise hatten weder die technische Affinität noch die subjektive Wichtigkeit des Datenschutzes Einfluss auf die Einstellung. Die Studie liefert damit wichtige Erkenntnisse für die weitere Erforschung und auch Praxis von Online-Beratung und vermag bisher widersprüchliche Befunde in dem Bereich zu erklären.

**Zusatzmaterial online:**

Zusätzliche Informationen sind in der Online-Version dieses Artikels (10.1365/s40896-021-00061-5) enthalten.

## Abstract (en)

Since the Corona pandemic the topic of “digitization” has also arrived in the world of consulting and counselling. However, online counselling is met with great scepticism by many practitioners. What exactly causes this negative attitude has been little studied. Previous studies have focused on either video therapy or the adoption of evidence-based practices. Therefore, the aim of the present cross-sectional study was to identify possible characteristics that positively or negatively influence attitudes toward online counselling compared with face-to-face counselling. To assess attitudes, a new instrument describing various characteristics of counselling was developed and administered via online questionnaire to 66 individuals from the fields of counselling, therapy, and coaching. A factor analysis yielded two different attitude dimensions for the instrument, each of which was predicted by different person characteristics in subsequent regression analyses. With respect to the dimension “core characteristics of consulting”, a more negative attitude is predicted only by higher professional experience. Regarding the dimension “general conditions of consultations”, a more positive attitude toward the online setting is revealed when the consultants identify themselves as women, have lower levels of professional experience, work in the field of coaching, or practice in urban locations. Interestingly, neither technical affinity nor subjective importance of privacy had an impact on attitudes. The study thus provides important insights for further research and also practice of online counselling and is able to explain previously contradictory findings in the field.

Die Digitalisierung und die damit einhergehende Integration der Technologie ist eines der am häufigsten diskutierten Themen im Bereich der Beratung, Therapie und des Coachings (Rees und Stone 2005; Simpson und Reid 2014b). Dabei haben die elektronischen Kommunikationsformen sowohl in der Medizin als auch in der Psychologie einen ausführlichen historischen Hintergrund (für einen Überblick siehe Sammons et al. 2020): Bereits 1879 wurden Telefone für die Betreuung von zu behandelnden Personen eingesetzt, um einen Besuch in der Praxis zu vermeiden. Erste Experimente, die die Anwendung der Telefonbehandlung im Vergleich zur Präsenzbehandlung untersuchten, stammen aus den 1960er-Jahren (für eine Übersicht siehe Nesbitt 2012). Die Weiterentwicklung des Internets in den 1990er-Jahren führte zu einem Aufschwung der Anwendung und Umsetzung von internetbasierten Formen der Therapie und Beratung, wie beispielsweise der E‑Mail-Beratung, und auch deren Erforschung entwickelte sich kontinuierlich weiter. Während die weitere Verbreitung dieser Formen in der Praxis langsam, aber stetig zunahmen, beschleunigten die im Zuge der Sars-Cov‑2 Pandemie verhängten Lockdowns in den Jahren 2020 und 2021 schließlich die Umstellung der Dienstleistungen von Coaches, Therapierenden und Beratenden auf Distanz-Beratung (WHO 2020). Dies hat zur Folge, dass Coachings, Beratungen und auch viele Therapien zu einem erheblichen Teil über elektronische Kommunikationsformen erfolgen mussten (Sammons et al. 2020).

Doch obwohl das Distanz-Setting mehrere Vorteile mit sich bringt (Backhaus et al. 2012; Eells et al. 2014; Schuster et al. 2018), auf Seiten der Ratsuchenden akzeptiert (Simpson und Reid 2014a), von manchen sogar präferiert (Wong et al. 2018) wird und zudem die Durchführbarkeit und Wirksamkeit von videobasierten Online-Therapien durch viele Studien belegt wurden (für Übersichten siehe z. B. Backhaus et al. 2012; Geissler und Metz 2012; Ghods und Boyce 2013), wird deren Einführung von Seiten der Beratenden, Therapierenden und Coaches skeptisch gesehen (Mitchel 2020; Smith et al. 2020). Aussagekräftige Studien zur Frage, warum diese Berufsgruppen eine solch negative Einstellung gegenüber dem Online-Setting haben, sind bislang Mangelware (Mitchel 2020; Schuster et al. 2018). Der vorliegende Beitrag möchte dazu beitragen, diese Forschungslücke ein Stück weit zu schließen und berichtet von einer Umfrage unter Beratenden, Coaches und Therapierenden zu deren Einstellung gegenüber Online-Beratung im Vergleich zur Präsenz-Beratung.

## Online-Beratung – eine Begriffsbestimmung

Für das Setting „Beratung über das Internet“ existiert eine Vielzahl unterschiedlicher Varianten und Bezeichnungen (Kupfer und Mayer 2019; Barak et al. 2009; Rochlen et al. 2004). Unter anderem findet man die Begriffe Teleberatung, virtuelles Coaching, E‑Coaching, Online-Counseling sowie Beratung im Netz (für eine Übersicht siehe Engelhardt und Storch 2013). Aufgrund der zahlreichen unterschiedlichen Bezeichnungen in der internationalen Fachliteratur wurden im deutschsprachigen Raum einige Systematisierungsversuche unternommen (Eichenberg und Küsel 2016; Mundelsee 2021). Gemeint ist in allen Fällen „eine computergestützte, medial über das Internet vermittelte, interaktive Beratung“ (Kupfer und Mayer 2019, S. 245). Dabei unterscheiden sich die Kommunikationsmittel nach der Anwendung und variieren je nach Präferenz. Demnach kann die Beratung u. a. schriftbasiert per E‑Mail, Chat und in Internetforen, sprachbasiert per Telefon, videobasiert per Videokonferenzen oder plattformbasiert durchgeführt werden (Eichenberg und Küsel 2016; Engelhardt und Storch 2013; Kühne und Hintenberger 2020). Die videobasierten Kommunikationsformen bilden dabei eine Annährung zur Präsenz-Beratung (Kupfer und Mayer 2019).

Eine weitere Unterscheidung ist hinsichtlich der Synchronität der Kommunikation möglich. Dabei kann die Beratung synchron beispielsweise per Videokonferenz, simultan per Chat oder asynchron per E‑Mail vorgenommen werden (Eichenberg und Küsel 2016; Barak et al. 2009). Auch verwandte Kontexte, wie die Online-Therapie oder das Online-Coaching, werden manchmal unter Online-Beratung subsummiert (Kupfer und Mayer 2019; Simpson und Reid 2014b).

Um der Vielzahl der unterschiedlichen Bezeichnungen Rechnung zu tragen, werden im nachfolgenden theoretischen Hintergrund die einzelnen Formen des untersuchten Online-Settings (also z. B. „Video-Therapie“) dann explizit genannt, wenn sie wichtig für das weitere Verständnis sind. In den anderen Fällen fassen wir unter dem Begriff „Online-Beratung“ alle oben genannten Formen zugunsten des Leseflusses zusammen. Die agierenden Fachkräfte, egal ob Beratende, Therapierende oder Coaches bezeichnen wir entsprechend als Beratende und Ratsuchende sowie beratende und ratsuchende Personen bzw. nennen ebenfalls explizit die Berufsgruppe.

## Vor- und Nachteile der Online-Beratung

Online-Beratung ermöglicht den beratenden und den ratsuchenden Personen einige Vorteile im Vergleich zur traditionellen Präsenz-Beratung (Backhaus et al. 2012; Eells et al. 2014; Schuster et al. 2018). In einschlägiger Fachliteratur werden dabei folgende Punkte häufig genannt und diskutiert: (1) Flexibilität und Niedrigschwelligkeit, da die Beratung im Internet als orts- und zeitungebundenes Angebot gilt (Kupfer und Mayer 2019), (2) leichter Zugang für Ratsuchende, denen ein adäquates Angebot in der näheren Umgebung fehlt (Barrett und Gershkovich 2014; Backhaus et al. 2012), (3) Anonymität und ein enthemmender Effekt, da die Möglichkeit besteht, seine Persönlichkeit zu verbergen, was wiederum die Offenheit und Ehrlichkeit der ratsuchenden Person erhöht (Kupfer und Mayer 2019; Kühne und Hintenberger 2020) und (4) betriebswirtschaftliche Vorteile durch Verringerung der Reisekosten und Vergrößerungen des Klientel (Backhaus et al. 2012; Schuster et al. 2018).

Bei all den skizzierten Vorteilen und dem daraus resultierenden Mehrwert werden in der Literatur auch Nachteile genannt, die mit der Online-Beratung verbunden sind. Dazu zählen u. a.: (1) Kanalreduktion, welche mit einem Mangel an visuellen Hinweisreizen einhergeht und zu Beginn einer Beratung Widerstände auslösen kann (Kupfer und Mayer 2019; Rees und Stone 2005), (2) Mangel an technischen Fähigkeiten (Kupfer und Mayer 2019; Overholser 2013), welches in einem unangenehmen und belastenden Gefühl der beratenden Personen resultiert, der ratsuchenden Person bei technischen Problemen oder Verbindungsabbrüchen nicht kompetent helfen zu können (Kupfer und Mayer 2019; Simpson et al. 2020), (3) Verbindungskosten durch anfallende Ausgaben für Online-Tools, Internetverbindung und technische Ausstattung (Simpson et al. 2020; Smith et al. 2020), (4) Bedenken bezüglich des Aufbaus der therapeutischen Beziehung (Simpson und Reid 2014a), (5) höherer Zeitaufwand durch erhöhte Einarbeitungszeit und Vorbereitungszeit (Schuster et al. 2018), (6) Störungsanfälligkeit durch schwankende Internetverbindungen (Simpson und Reid 2014a; Smith et al. 2020), (7) kaum Schulungsangebote (Simpson et al. 2020) und (8) Bedenken hinsichtlich des Datenschutzes (Eells et al. 2014; Kupfer und Mayer 2019).

Trotz der Falsifizierung einiger dieser Nachteile durch diverse Studien und der Entwicklung neuer Ratgeber und Tools für die Online-Beratung gibt es vor allem auf Seiten der Beratenden noch viele Vorbehalte. So zeigt beispielsweise das Review von Smith et al. (2020), dass beratende Personen das Online-Setting als störend, komplex und minderwertig im Vergleich zum Präsenz-Setting einschätzen (siehe auch Mitchel 2020; Simpson und Reid 2014b). Gleichzeitig wird das Online-Setting auf der Seite der Ratsuchenden akzeptiert und von manchen sogar präferiert. In einer Umfrage unter Studierenden gaben beispielsweise 35 % der Teilnehmenden an, dass sie Online-Beratung in Anspruch nehmen würden, aber nicht an Beratung in Präsenz teilnehmen würden (Wong et al. 2018). Während es für das Online-Beratungssetting jedoch an empirischen Studien fehlt, die den Ursprung der Vorbehalte der Beratenden untersuchen (Mitchel 2020; Schuster et al. 2018), geben Untersuchungen zu verwandten Kontexten einige Hinweise darauf.

## Einstellung gegenüber verwandten Kontexten

Eine Einstellung ist eine „psychische Tendenz, die dadurch zum Ausdruck kommt, dass man ein bestimmtes Objekt mit einem gewissen Grad an Zuneigung oder Ablehnung bewertet“ (Eagly und Chaiken 1993, S. 1). Neben der subjektiven Norm und der wahrgenommenen Verhaltenskontrolle ist die Einstellung gegenüber einem Einstellungsobjekt ein wesentlicher Einflussfaktor auf das Verhalten einer Person (Ajzen 1991). Studien zur Einstellung gegenüber Einstellungsobjekten, die der (Einführung von) Online-Beratung ähnlich sind, liefern einige Hinweise auf mögliche Faktoren, die auch die Einstellung gegenüber der Online-Beratung beeinflussen könnten.

In zwei Studien untersuchte Aarons beispielsweise individuelle Merkmale von Mitarbeitenden des klinischen Dienstes bei der Einführung evidenzbasierter Praktiken (engl. EBP, Evidence Based Practice; Aarons 2004; Aarons et al. 2010). Bei EBP handelt es sich um ein Konzept im klinischen Bereich, bei dem davon ausgegangen wird, dass berufliche Praktiken auf wissenschaftlichen Erkenntnissen beruhen sollen und Fachkräfte diejenige Methode zur Behandlung anwenden soll, die laut Forschung am effektivsten ist. Online-Beratungen können als solche EBP eingeschätzt werden (Sammons et al. 2020). Die Ergebnisse der ersten Studie (*N* = 322) zeigen, dass eine höhere positive Einstellung zur Einführung von EBP zur wahrscheinlicheren Nutzung bei älteren Praktizierenden beobachtet werden konnte (Aarons 2004). Zur Bestätigung der Ergebnisse wurde eine zweite Studie an Mitarbeitenden des klinischen Dienstes (*N* = 1089) durchgeführt, wobei einige Erkenntnisse repliziert werden konnten (Aarons et al. 2010). Demnach führen ein höheres Alter, die Identifikation mit dem weiblichen Geschlecht und der weißen Rasse sowie weniger Jahre Berufserfahrung zu einer positiveren Einstellung gegenüber neuer Praxisansätze.

Daran anlehnend untersuchten Perle et al. (2014) die Einstellung psychologischer Fachkräfte (*N* = 717) gegenüber videogestützter Online-Psychotherapie (kurz Video-Therapie). Die Ergebnisse verdeutlichen, dass Datenschutzprobleme, Bedenken hinsichtlich der Kundensicherheit, rechtliche Bedenken und die mangelnde Ausbildung dazu führen, dass psychologische Fachkräfte eine negativere Einstellung bezüglich der Video-Therapie aufweisen und deren Einführung vermeiden (Perle et al. 2014).

Pierce et al. (2020) griffen diese Erkenntnisse auf und ermittelten anhand der Einstellungen von US-Psychologen zur Video-Therapie demographische, organisatorische und klinische Prädiktoren, welche die Verwendung der Video-Therapie vorhersagten. Dabei wurde angenommen, dass eine positive Einstellung die Verwendung und eine negative Einstellung die Ablehnung der Video-Therapie verursacht, wobei für die vorliegende Studie nur die Einstellungseinschätzungen relevant ist. Die Ergebnisse der Studie (*N* = 1799) zeigen, dass die Anzahl durchgeführter Schulungen und die geographische Lage der Praxis entscheidend für die Einstellung und weiterführend für die Verwendung der Video-Therapie sein kann. Demnach ist es wahrscheinlicher, dass Fachkräfte eine positivere Einstellung in Bezug auf die Video-Therapie aufweisen und diese verwenden, wenn sie in einem urbanen Gebiet praktizieren und die Annahme vertreten, dass sie ausreichend in der Anwendung der Video-Therapie geschult sind. Zusätzlich wurden in ihren Arbeiten das Alter und das weibliche Geschlecht als mögliche Prädiktoren untersucht. Im Gegensatz zu den Studien von Aarons (Aarons 2004; Aarons et al. 2010) führten die Identifizierung als Frau und das Alter bei Pierce et al. (2020) zu keiner Einstellungsveränderung hinsichtlich der Video-Therapie und zählten demnach nicht zu den signifikanten Prädiktoren für die Verwendung.

Zusätzlich konnte in den Studien ein weiterer potenzieller Prädiktor identifiziert werden. Die Praxis der Video-Therapie erfordert spezielle Fähigkeiten und Kenntnisse, um praktische Herausforderungen zu bewältigen (Pierce et al. 2020). Simpson et al. (2020) fassen in ihren Arbeiten spezifische Voraussetzungen zusammen, die für die Umsetzung der Video-Therapie entscheidend sein sollten. Dazu zählen die Autoren vor allem die Technikbereitschaft der Therapierenden und der ratsuchenden Person (Simpson et al. 2020). Die Technikbereitschaft kann dabei als dreifaktorielles Konstrukt mit den Facetten Technikakzeptanz, Technikkompetenz- und Technikkontrollüberzeugungen verstanden werden. Die daraus resultierende Akzeptanz und das Interesse an der Technologie wirken sich positiv auf das Wissen und die Anwendung aus (Karrer et al. 2009; Neyer et al. 2016). Somit kann ein Mangel an Technikbereitschaft, welcher sich in fehlender Medienkompetenz und -akzeptanz äußert, zu einer negativeren Einstellung und somit zur Ablehnung des Online-Settings führen (Pierce et al. 2020).

## Ziel der vorliegenden Studie

Zur Frage, warum Beratende dem Online-Setting so skeptisch gegenüberstehen, fehlt es bislang an aussagekräftigen Studien (Mitchel 2020; Schuster et al. 2018). Bisherige Untersuchungen bezogen sich entweder auf Video-Therapien oder auf die Einführung evidenzbasierter Praktiken. Zudem wurde bislang nur die Einstellung gegenüber dem Online-Setting losgelöst vom Präsenz-Setting untersucht. Ziel der vorliegenden Studie war es daher, Faktoren zu ermitteln, die anlehnend an die Erkenntnisse aus den anderen Kontexten die Einstellung von beratenden Personen gegenüber dem Online-Setting im Vergleich zum Präsenz-Setting prädizieren. Anlehnend an die beschriebenen Erkenntnisse aus der Beforschung zur Video-Therapie und zur Einführung evidenzbasierter Praktiken werden folgende Hypothesen aufgestellt:

### (H1)

Zu einer positiveren Einstellung der beratenden Personen gegenüber dem Online-Setting im Vergleich zum Präsenz-Setting führen (a) ein höheres Alter, (b) eine geringere Berufserfahrung, (c) die Identifikation als Frau (im Gegensatz zur Identifikation als Mann), (d) eine urbane Lage der eigens oder kollegial genutzten Praxisräume (im Gegensatz zu einer ruralen Lage), (e) eine höhere Anzahl an absolvierten Schulungen für die Online-Beratung, (f) eine höhere Technikbereitschaft und (g) eine höhere Datenschutzwichtigkeit.

Darüber hinaus wurde aufgrund bislang noch nicht vorliegender Befunde folgende explorative Forschungsfrage formuliert:

### (F1)

Hat die Perspektive aus den verschiedenen Berufsrollen der beratenden Personen (Beratung, Coaching, Therapie) einen Einfluss auf deren Einstellung bezüglich des Online-Settings?

## Methode

### Stichprobe & Design

Zielgruppe der Studie waren beratende Personen mit abgeschlossener Ausbildung. Im Sinne eines Querschnittsdesigns füllten 99 Teilnehmende einmalig einen Online-Fragebogen aus, wovon 33 (33 %) aufgrund vorab definierter Kriterien (unzureichender Abschluss, nicht ausgefüllte Einwilligungserklärung, unvollständig ausgefüllte Fragebögen) ausgeschlossen wurden. Die finale Stichprobe besteht somit aus 66 Personen. Dies erfüllt die a priori ermittelte notwendige Stichprobengröße von *N* = 43 (Poweranalyse mit α‑Fehler-Wahrscheinlichkeit = 0,05, Power = 0,80 und einer erwarteten Effektstärke von ƒ^2^ = 0,15). Dennoch war es zur Verbesserung der Repräsentativität das ursprüngliche Ziel, eine größere Stichprobe zu erreichen. Da dies nicht gelang, haben wir in Tab. 1 viele deskriptive Merkmale der Stichprobe aufgeführt und auf deskriptiver Ebene mit der umfangreichen RAUEN Coaching-Marktanalyse (2020) verglichen. Ein typisches Merkmal der Beratungs-Branche ist demnach ein leichter Überhang des weiblichen Geschlechts. Zusätzlich konnte in den Analysen ein Durchschnittsalter von 53,75 Jahren und eine durchschnittliche Berufserfahrung als beratende Person von 12,59 Jahren ermittelt werden (Rauen 2020). Diese Kennzeichen finden sich auch in der vorliegenden Stichprobe. Auch wenn dadurch die Einschränkung der geringen Stichprobengröße bei den vorliegenden Ergebnissen bestehen bleibt, spricht dieser Vergleich zumindest in den genannten Aspekten für die Repräsentativität der vorliegenden Stichprobe.

**Table 1:** 

Merkmal	*n* (%)
*Geschlecht*	
Weiblich	52 (78,78)
Männlich	13 (19,70)
Divers	1 (1,52)
*Eigene Praxisräume*	
Ja	30 (45,45)
Nein	19 (28,79)
Kollegial	17 (28,76)
*Praxisort*	
Urban	29 (61,70)
Rural	18 (38,30)
Sonstiges/keine Angabe	–
*Berufsrolle*^*a*^	
Beratung	54 (81,82)
Coaching	46 (69,70)
Therapie	26 (39,39)
Sonstiges	13 (19,70)
*Nutzung von Online-Beratung*	
Ja	59 (89,40)
Nein	7 (10,60)
*Teilnahme an einer Schulung zum Thema Online-Beratung/-Therapie*	
Ja	53 (80,30)
Nein	13 (19,70)

*N* = 66

^a^Mehrfachauswahl möglich

### Durchführung

Die Datenerhebung wurde mittels einer Online-Umfrage durchgeführt. Die Akquise potenzieller Probanden fand über unterschiedliche Kanäle statt, u. a. Facebook-Gruppen und Newsletter einiger Dachverbände.

Zu Beginn der Erhebung wurden die Teilnehmenden grob über die Ziele der Erhebung aufgeklärt und ihre Einwilligung zur Datenverarbeitung eingefordert. Es folgten Fragen zur Einstellung der Probanden gegenüber Online-Beratung (eigene Items), zur Technikbereitschaft (Neyer et al. 2016) und der technischen Affinität (Karrer et al. 2009) sowie zur Wichtigkeit des Datenschutzes (eigene Items). Abgeschlossen wurde der Fragebogen mit der Erhebung demographischer Daten und anderer relevanter Merkmale (vgl. Tab. 1).

Die Beteiligung an der Befragung war freiwillig, unbelohnt und ein Abbruch war zu jederzeit möglich. Die Erhebung entsprach den ethischen Standards des nationalen Forschungsausschusses und ihren späteren Änderungen oder vergleichbaren ethischen Standards.

### Erhebungsinstrumente

#### Einstellungen

In Anlehnung an Aarons (2004), Aarons et al. (2010), Perle et al. (2014) sowie Pierce et al. (2020) wurde eine eigene Skala zur expliziten Erfassung der Einstellung gegenüber Online-Beratung entwickelt. Hierzu wurden anlehnend an einschlägige Fachliteratur wesentliche Merkmale von Beratungen, Therapien und Coachings herausgearbeitet und anschließend den einzelnen Komponenten der Einstellung gemäß dem Drei-Komponenten-Modell nach Rosenberg und Hovland (1960) zugeordnet (siehe Online-Ressourcen 1).

Um der Fragestellung gerecht zu werden, ob sich Unterschiede in den Einstellungen der beratenden Personen ergeben, wenn das Online-Setting dem Präsenz-Setting gegenübergestellt wird, erfolgte die Erfassung der Einstellung mithilfe einer 7‑stufigen bipolaren Skala im vergleichenden Format. Auf die Frage „Wie schätzen Sie die Online-Beratung/-Therapie im Vergleich zur Präsenz-Beratung/-Therapie in Bezug auf …“ sollten die einzelnen Merkmale von Beratungen, Therapien und Coachings anhand der Antwortoptionen 1 = „deutlich besser in Präsenz“, 4 = „gleich gut“ sowie 7 = „deutlich besser online“ eingeschätzt werden.

Die Zuordnung zu den drei Komponenten von Einstellungen (Rosenberg und Hovland 1960) diente der Orientierung bei der Entwicklung und wurde anhand der vorliegenden Stichprobe mittels einer explorativen Faktorenanalyse überprüft. Zur Bestimmung der Faktorenanzahl wurde das Kaiser-Guttmann-Kriterium erweitert durch die Parallelanalyse nach Horn (1965) angewandt. Die Analyse ergab zwei Faktoren, sodass das Drei-Komponenten-Modell (Rosenberg und Hovland 1960) aufgegeben wurde. In der rotierten Lösung (oblique Rotation, Cut-off-Wert = 0,40; Field et al. 2012) luden neun Items auf Faktor 1, die inhaltlich als solche Merkmale zusammengefasst werden können, die den Kern von Beratungen, Therapien und Coachings bzw. notwendige Erfolgsfaktoren betreffen (im Folgenden kurz als „Kernmerkmale von Beratungen“ bezeichnet; Beispiel-Items: „professionelle Unterstützung“, „Coach-Klient-Beziehung“, „Gefühle wahrnehmen“, „therapeutischer/beraterischer Nutzen“), auf Faktor 2 luden dagegen sieben Items, die inhaltlich eher zu den Rahmenbedingungen von Beratungen, Therapien und Coachings bzw. deren hinreichenden Erfolgsfaktoren zählen (im Folgenden kurz als „Rahmenbedingungen von Beratungen“ bezeichnet; Beispiel-Items: „Anonymität“, „finanzieller Aufwand“, „Flexibilität“). Das Item „Qualität“ lud auf beiden Faktoren und wurde deshalb ausgeschlossen.

Die aus den Mittelwerten der jeweiligen Items gebildeten beiden Subskalen weisen gute interne Konsistenzen auf (Cronbachs α der Subskala „Kernmerkmale“ = 0,89; Cronbachs α der Subskala „Rahmenbedingungen“ = 0,82). Demnach kann die Reliabilität dieses neuen Instrumentes als gegeben angenommen werden. Aufgrund der Neuartigkeit des Instrumentes war die Bestimmung der Übereinstimmungsvalidität und der Konstruktvalidität nicht möglich. Jedoch können aufgrund der Ableitung der Items aus einschlägiger Fachliteratur Inhaltsvalidität und Augenscheinvalidität angenommen werden.

#### Datenschutz

Zur Erfassung der Wichtigkeit des Datenschutzes wurden Aussagen aus einem Leitfaden von Hörmann et al. (2019) verwendet, in dem die Anforderungen an digitale Medien in der Beratung hinsichtlich des Datenschutzes zusammengefasst werden (siehe Online-Ressourcen 2). Die Aussagen konnten mittels einer fünfstufigen Likert-Skala (1 = „Trifft gar nicht“ zu; 5 = „Trifft voll zu“) beantwortet werden. Dabei weist ein hoher Wert auf eine höhere persönliche Wichtigkeit des Datenschutzes hin. Die interne Konsistenz dieser Skala war zufriedenstellend (Cronbachs α = 0,62).

#### Technikbereitschaft

Zur Erfassung der Technikbereitschaft wurde zum einen die Kurzskala von Neyer et al. (2016) verwendet. Diese dient dazu, die Akzeptanz und die Technikkompetenz der Befragten zu erfassen. Dazu wurden einzelne Aussagen präsentiert, welche die Befragten auf einer fünfstufigen Likert-Skala (1 = „Trifft gar nicht“ zu; 5 = „Trifft voll zu“) einschätzen sollten. Dabei weist ein hoher Wert auf eine höhere Technikbereitschaft hin. Die Validierung der Skala erfolgte anhand von drei Stichproben (Cronbachs α = 0,74 bis 0,84), wobei Hinweise auf Kriteriums- und Konstruktvalidität ermittelt werden konnten. Zum anderen wurden drei Items des TA-EG (Karrer et al. 2009) zur Erfassung der Technikaffinität eingesetzt, um die persönliche Meinung zu verschiedenen Aspekten elektronischer Geräte aufzugreifen (siehe Online-Ressourcen 3). Cronbachs α lag in dieser Studie für die beiden zusammengefassten Skalen bei 0,86.

#### Demographische Daten

Zudem wurden die Befragten gebeten, demographische Daten über sich selbst (Alter, identifiziertes Geschlecht, Berufserfahrung), Informationen über ihre Arbeit (Berufsrolle, Praxisort), über die Anzahl an absolvierten Schulungen und Trainingsmaßnahmen für die Beratung im Online-Setting sowie über die persönliche Nutzung der Online-Beratung, zur Verfügung zu stellen. Dazu wurden zum Teil auch offene Fragen gestellt, die nicht in die folgenden Auswertungen eingeflossen sind.

### Datenanalyse

Alle Analysen wurden mit RStudio (Version 4.1.0) unter zu Hilfenahme der Pakete „psyc“, „car“, „stats“, „QuantPsyc“, „leaps“ durchgeführt. Es wurde sichergestellt, dass die erhobenen Daten die Anforderungen der einzelnen Pakete erfüllen.

Fehlende Werte (in der vorliegenden Stichprobe 1,15 % aller in die nachfolgenden Analysen einfließenden Daten) wurden unter Zuhilfenahme des Verfahrens der multiplen Imputation ersetzt (Allison 2000; Hox 1999). Aufgrund der unterschiedlichen Skalierungen wurden alle Variablen vor den Analysen *z*-standardisiert. Zur Bestimmung möglicher Prädiktoren wurde eine Regressionsanalyse durchgeführt (Alpha-Level von 0,05). Angesichts des explorativen Charakters der Studie und der zahlreichen Prädiktoren (Berufserfahrung, identifiziertes Geschlecht, Technikbereitschaft, Datenschutzwichtigkeit, Praxisort, Anzahl der Schulungen, Anzahl der Online-Beratungen und Berufsrolle), wurde das von Lumley (2020) entwickelte R‑Paket „leaps“ verwendet. Das Paket führt eine erschöpfende Suche nach den besten Prädiktoren durch, wobei ein sogenannter Branch-and-Bound-Algorithmus verwendet wird. Der Algorithmus wählt aus allen Prädiktoren jene Kombination aus, die zum besten angepassten R‑Quadrat führt.

## Ergebnisse

### Voranalysen

#### Multikollinearität

Zur Bestimmung möglicher Multikollinearität wurde eine Korrelationsanalyse durchgeführt (siehe Tab. 2). Es konnte eine hohe Korrelation (*r* = 0,66) zwischen dem Alter und der Berufserfahrung ermittelt werden, weshalb nur die Berufserfahrung in die nachfolgenden Untersuchungen miteinbezogen und Hypothese 1a nicht mehr weiterverfolgt wurde.

**Table 2:** 

Variable	*M (SD)*	1	2	3	4	5	6	7
1. Einstellung ggü. Kernmerkmalen^a^	3,46 (0,79)	–	–	–	–	–	–	–
2. Einstellung ggü. Rahmenbedingungen^a^	4,28 (1,08)	0,60***	–	–	–	–	–	–
3. Technische Affinität^b^	3,57 (0,62)	−0,04	−0,04	–	–	–	–	–
4. Datenschutzwichtigkeit^b^	4,19 (0,75)	−0,05	−0,11	0,23	–	–	–	–
5. Anzahl der Online-Beratungen	44,25 (55,46)	−0,18	−0,12	0,08	0,19	–	–	–
6. Anzahl der Schulungen	3,55 (2,69)	0,09	0,07	−0,10	0,00	0,06	–	–
7. Alter	47,70 (11,58)	−0,08	−0,17	0,01	0,10	0,19	0,06	–
8. Berufserfahrung	11,39 (9,34)	−0,26*	−0,25*	−0,11	−0,02	0,36**	0,19	0,66***

*N* = 66

*p < 0,05, **p < 0,01, ***p < 0,001

^a^7‑stufiges Antwortformat (1 bis 7)

^b^5‑stufiges Antwortformat (1 bis 5)

#### Personenausschluss

Da sich beim identifiziertes Geschlecht nur eine Person der Kategorie „divers“ und bei der Berufsrolle nur eine Person ausschließlich der Kategorie „sonstiges“ zugeordnet hatte, wurden diese aus Repräsentativitätsgründen von den nachfolgenden Analysen ausgeschlossen.

### Hauptanalysen

Zur Überprüfung von Hypothesen 1b bis 1g sowie der explorativen Forschungsfrage 1 wurden zwei multivariate Regressionsanalysen mit den beiden Einstellungssubskalen als jeweiliges Kriterium sowie mit der besten durch den „leaps“-Algorithmus ermittelten Prädiktorenkombination durchgeführt.

#### Einstellungssubskala „Kernmerkmale von Beratungen“

Für die Einstellung gegenüber den Kernmerkmalen von Beratungen konnten das identifizierte Geschlecht, die Berufserfahrung und die jeweilige Berufsrolle als beste Prädiktorenkombination ermittelt werden (siehe linke Seite der Tab. 3). Das Modell wird signifikant (*F*(5,59) = 3,11, *p* = 0,01). Das korrigierte *R*^2^ beträgt 0,14 und entspricht nach Cohen (1992) einer mittleren Effektstärke (*f* = 0,16). Entsprechend Hypothese 1b konnte ein signifikanter negativer Effekt der Berufserfahrung auf die Einstellung gegenüber den Kernmerkmalen von Beratungen ermittelt werden. Bezüglich der Hypothesen 1c bis 1g und der explorativen Forschungsfrage konnten keine signifikanten Unterschiede gefunden werden, wobei bei der Betrachtung der Berufsrolle „Coaching“ die Tendenz zu einer positiveren Einstellung gegenüber den Kernmerkmalen von Beratung ermittelt werden konnte.

**Table 3:** 

	Einstellung ggü. Kernmerkmalen				Einstellung ggü. Rahmenbedingungen			
	β	*SE*	*t*	*p*	β	*SE*	*t*	*p*
Intercept	−0,80	0,50	−1,62	0,11	−0,54	0,46	−1,18	0,24
Identifiziertes Geschlecht^a^	0,31	0,30	1,02	0,31	0,60	0,28	2,14	0,04*
Berufserfahrung	−0,34	0,13	−2,67	0,01*	−0,41	0,13	−3,23	0,002**
Berufsrolle „Coaching“^b^	0,52	0,31	1,68	0,09^†^	0,58	0,29	2,03	0,04*
Berufsrolle „Beratung“^b^	0,37	0,30	1,23	0,22	0,03	0,28	0,11	0,92
Berufsrolle „Therapie“^b^	−0,29	0,27	−1,07	0,29	−0,48	0,27	−1,80	0,08^†^
Praxisort^c^	–	–	–	–	0,45	0,22	2,00	0,05^†^
Anzahl Beratungen online	–	–	–	–	0,16	0,13	1,22	0,23

*N* = 64. β gibt die standardisierten Regressionsgewichte an

^†^*p* < 0,10; *p < 0,05; **p < 0,01

^a^Kodierung 0 = männlich, 1 = weiblich

^b^Kodierung 0 = der Berufsrolle nicht zugehörig, 1 = der Berufsrolle zugehörig

^c^Kodierung 0 = rural, 1 = urban

#### Einstellungssubskala „Rahmenbedingungen von Beratungen“

Das identifizierte Geschlecht, der Praxisort, die Berufserfahrung, die jeweilige Berufsrolle und die Anzahl der Online-Beratung konnten als beste Prädiktorenkombination für die Einstellung gegenüber den Rahmenbedingungen von Beratungen ermittelt werden (siehe rechte Seite der Tab. 3). Das Modell wird signifikant (*F*(7,57) = 3,79, *p* = 0,002). Cohens *f* beträgt 0,30 (korrigiertes *R*^2^ = 0,23) und spiegelt eine mittlere Effektstärke wider (Cohen 1992). In Übereinstimmung mit Hypothesen 1b, 1c und 1d sagen eine höhere Berufserfahrung eine negativere Einstellung und die Identifikation mit dem weiblichen Geschlecht sowie eine urbane Lage des Praxisortes eine positivere Einstellung gegenüber den Rahmenbedingungen von Beratungen vorher. Zugunsten der explorativen Forschungsfrage deuten die Ergebnisse auf einen Einfluss der Berufsrolle auf die Einstellung der beratenden Personen gegenüber den Rahmenbedingungen von Beratungen hin. Während Personen in der Berufsrolle „Beratung“ diesbezüglich keine signifikant unterschiedliche Einstellung zeigen, sind sie in der Berufsrolle „Coaching“ den Rahmenbedingungen gegenüber signifikant positiver und in der Berufsrolle „Therapie“ marginal signifikant negativer eingestellt. Bezüglich der Hypothesen 1e, 1f und 1g konnten keine signifikanten Effekte festgestellt werden.

## Diskussion

Ziel der Studie war es, mögliche Prädiktoren für Einstellung von Beratenden gegenüber der Online-Beratung im Vergleich zur Präsenz-Beratung zu ermitteln und damit einen Teil dazu beizutragen, die bestehende Forschungslücke zu schließen. Da ein neues Instrument zur Erfassung der Einstellung entwickelt werden musste, dessen weitere Validierung aussteht, und die Stichprobe nicht die erhoffte Größe für eine hohe Repräsentativität erreichte, konnte zur Schließung der bestehenden Forschungslücke nur ein kleiner Beitrag geleistet werden. Dennoch ergeben sich interessante Befunde für die weitere Erforschung und Praxis von Online-Beratung.

Die Voranalysen des neuen Instrumentes ergaben zwei unterschiedliche Dimensionen in Bezug auf die Merkmale von Beratung, Therapie und Coaching: Auf der einen Seite solche Eigenschaften, die sich auf den Kern von Beratungen beziehen bzw. notwendige Erfolgsfaktoren für diese darstellen (in der Studie als Kernmerkmale von Beratung bezeichnet) und auf der anderen Seite Aspekte, die eher zu den Rahmenbedingungen von Beratungen zählen bzw. als hinreichende Erfolgsfaktoren gefasst werden können (Rahmenbedingungen von Beratung). Die Beratenden, die an der Umfrage teilgenommen hatten, unterscheiden sich in ihrer Einstellung gegenüber dem Online-Setting somit offensichtlich zwischen diesen beiden Dimensionen. Auch wenn die Unterteilung aufgrund der geringen Teilnehmerzahl sowie der Neuentwicklung des Instruments mit Vorsicht zu behandeln ist und es weiterer Forschung zu dessen Validierung bedarf, ist dieses Ergebnis dennoch bemerkenswert. Insbesondere auch deshalb, da die nachfolgenden Analysen in Bezug auf die beiden Faktoren ein uneinheitliches Bild ergaben und so das Potenzial haben, einige der Widersprüche in der bisherigen Befundlage aufzuklären.

Interessant sind bereits die deskriptiven Statistiken, die bei den Rahmenbedingungen eine leichte Präferenz des Online-Settings gegenüber dem Präsenz-Setting zeigen, jedoch eine hohe Streuung aufweisen, wohingegen bei den Kernmerkmalen das Präsenz-Setting präferiert wird. Bei beiden Maßen liegt der Mittelwert der Skala jedoch innerhalb von einer Standardabweichung. Dies ist für die folgenden Betrachtungen relevant, da es nahelegt, dass weder das eine noch das andere Setting von den Teilnehmenden der Studie besonders bevorzugt wird. Die nachfolgenden Ergebnisse können somit als Hinweise auf die Einstellung gegenüber beiden Settings von einem neutralen Standpunkt aus interpretiert werden.

Während das identifizierte Geschlecht, die Berufsrolle sowie die Lage des Praxisortes bei der Einstellung gegenüber den Kernmerkmalen von Beratungen keine oder wie bei der Berufsrolle „Coaching“ nur eine geringe Rolle spielten, waren es bei den Rahmenbedingungen genau diese Prädiktoren, die einen signifikanten Einfluss hatten: die Identifikation als Frau, die urbane Lage des Praxisortes sowie die Perspektive aus der Berufsrolle „Coaching“ sagten eine positivere Einstellung gegenüber den Rahmenbedingungen von Beratungen im Online-Setting im Vergleich zu Präsenz-Beratungen vorher.

Die Aufteilung in die beiden Einstellungsfaktoren vermag somit die bislang gefundenen Widersprüche in Bezug auf das Geschlecht erklären: Passend zu Aarons (2004) und Pierce et al. (2020), die die Identifizierung als Frau in Bezug auf die Einführung und Nutzung neuer evidenzbasierter Praktiken und Video-Therapie nicht als statistisch bedeutsamen Prädiktor ermitteln konnten, spielte das Geschlecht in Bezug auf die Kernmerkmale von Beratungen in der vorliegenden Studie ebenfalls keine Rolle. Dagegen führte die Identifizierung als Frau im Vergleich zur Identifizierung als Mann bei Aarons et al. (2010) zu einer wahrscheinlicheren Nutzung neuer Praxisansätze, was somit mit den Befunden zu den Rahmenbedingungen in der vorliegenden Studie einhergeht: Personen, die sich mit dem weiblichen Geschlecht identifizieren, sehen offenbar im Online-Setting die Chance, zumindest einige der Rahmenbedingungen, wie beispielsweise den eigenen finanziellen Aufwand oder die Flexibilität zu verbessern. Ein möglicher Erklärungsansatz für diesen Befund findet sich in der Literaturrecherche von Edding (2014) zu den Wünschen einer Frau in Bezug auf ihre Arbeit. Die Erkenntnisse der Recherche legen nahe, dass Frauen im Vergleich zu Männern sicherheitsorientierter eingestellt sind und die Vereinbarkeit von Beruf und Familie sowie die Weiterentwicklungsmöglichkeiten wesentliche Kriterien für die Ausübung eines Berufes sind. Im Kontext von Online-Beratungen vermögen Frauen genau diese Wünsche zu realisieren. Aufgrund der Zeit- und Ortsunabhängigkeit sowie der gesteigerten Flexibilität der Online-Beratung durch wegfallende Reisezeiten und der möglichen Weiterentwicklung im Bereich Medienkompetenz scheint das Online-Setting eine potenzielle Antwort auf die Bedürfnisse und Anforderungen von Frauen zu sein. Demnach könnte das Online-Setting für Frauen in Bezug auf einige Rahmenbedingungen der Beratung zufriedenstellender sein als beim Präsenz-Setting. Die Unterscheidung von Einstellungsdimensionen sollte somit in zukünftigen Studien angewandt werden, um weitere Klarheit in Bezug auf das identifizierte Geschlecht zu erhalten.

Auch der Praxisort konnte bei der Einstellung gegenüber den Rahmenbedingungen als statistisch bedeutsamer Prädiktor bestimmt werden und bestätigt damit die Ergebnisse von Pierce et al. (2020), dass Beratende in urbanen Gebieten zumindest in Bezug auf die Rahmenbedingungen von Beratung gegenüber dem Online-Setting positiver eingestellt sind als Beratende, die in ruralen Gebieten leben. Dieses Ergebnis könnte beispielsweise auf die fehlenden Mittel zur Umsetzung der Digitalisierung in ruralen Gebieten zurückzuführen sein (Pierce et al. 2020).

Entsprechend bisheriger Befunde in verwandten Kontexten (Aarons et al. 2010) sagte eine höhere Berufserfahrung in der vorliegenden Studie sowohl eine negativere Einstellung gegenüber den Kernmerkmalen wie auch den Rahmenbedingungen von Beratung vorher. Dazu passen Untersuchungen, die die Berufserfahrung in Zusammenhang mit beruflichen Veränderungen bringen. Das Ausführen einer bestimmten Tätigkeit führt zu einer stabilen Routine und Automatisierung bestimmter Handlungen, je länger diese Tätigkeiten ausgeführt werden, somit also auch mit zunehmender Berufserfahrung (French und Sternberg 1989; Hesketh et al. 1989). Ändern sich aber die Arbeitsbedingungen, wie z. B. während der Corona-Pandemie geschehen, aber auch im Zug von Change-Prozessen in Unternehmen, müssen diese Routinen und Automatismen manches Mal gegen ihren Willen von den betreffenden Personen abgelegt werden (Molter et al. 2013). Vor diesem Hintergrund passen die in der vorliegenden Studie gefundenen negativeren Einstellungen in Abhängigkeit von einer höheren Berufserfahrung somit zu den Ergebnissen der Untersuchung von Niessen et al. (2010), die die Berufserfahrung als Moderator für den negativen Zusammenhang zwischen Alter und Personen-Job-Passung nach organisationalen Veränderungen ermitteln konnten.

Wichtig ist jedoch hierbei anzumerken, dass das Alter und die Berufserfahrung in der vorliegenden Studie hoch miteinander korrelierten. Um Multikollinearität zu vermeiden, wurde deshalb nur die Berufserfahrung in die Analysen mit aufgenommen. Damit kann jedoch davon ausgegangen werden, dass auch ein höheres Alter mit einer negativeren Einstellung gegenüber beiden Faktoren einhergeht und dies damit bisherigen Befunden verwandter Kontexte widerspricht (Aarons 2004; Aarons et al. 2010; Pierce et al. 2020). Ursächlich für diese negativere Einstellung könnten die unterschiedliche generationsspezifische Wahrnehmung von Technik und die Verhaltens- und Leistungsunterschiede zwischen jüngeren und älteren Personen sein (Jakobs et al. 2008; Lödige-Röhrs 1995). Dabei scheint das Erleben von Technik in der ersten und zweiten Lebensphase und die dort erworbene Erfahrung im Umgang mit Technik wesentlich zu sein. Anlehnend an die Ergebnisse von Jakobs et al. (2008) wollen jüngere Personen technische Geräte ausschließlich konsumieren und ältere Personen die mechanisch-haptische Funktionsweise des Gerätes verstehen. Dieses gewünschte Verständnis lässt sich aber kaum auf die digitale Technik übertragen und führt zu ungünstigen Attributionsmustern und Vermeidungsstrategien (Jakobs et al. 2008; Lödige-Röhrs 1995). Zusammengenommen entsprechen diese Erkenntnisse dem Altersstereotyp, dass jüngere Personen neue Technologien wahrscheinlicher einführen und nutzen als ältere Personen (Lödige-Röhrs 1995, Pierce et al. 2020).

In dieser Hinsicht geben jedoch nicht nur die statistisch bedeutsamen Prädiktoren der vorliegenden Studie ein aufschlussreiches Bild ab. So hatten interessanterweise weder die subjektive Wichtigkeit des Datenschutzes noch die technische Affinität einen Einfluss auf die Einstellung der Teilnehmenden gegenüber Online-Beratung. Dies steht im Widerspruch zu den Erkenntnissen von Perle et al. (2013) und Simpson et al. (2020), die den Mangel an technischen Fähigkeiten und die Bedenken hinsichtlich des Datenschutzes als Barrieren zur Einführung des Online-Settings feststellten. Dies könnte ein Resultat der Weiterentwicklung der verwendeten Tools zur Online-Beratung sein, da einige von diesen während der Pandemie benutzerfreundlicher und datenschutzkonformer gestaltet wurden.

Des Weiteren deuten die Ergebnisse darauf hin, dass die Einstellung gegenüber Online-Beratung nicht von der Anzahl der absolvierten Trainings- oder Schulungsmaßnahmen abhängig ist. Es könnte zwar sein, dass die vorliegende Stichprobe in diesem Aspekt verzerrt ist und viele Personen enthält, die auf Schulung verzichten, weil sie sehr technikaffin sind. Der schwach negative, jedoch nicht signifikante Zusammenhang zwischen Technikaffinität und Anzahl der besuchten Schulungen spricht allerdings gegen diese Hypothese (vgl. Tab. 2). Das Ergebnis ist auch deshalb unerwartet, da der derzeitige Forschungsstand nahelegt, dass Trainings und Schulungen zu einer Verbesserung der Einstellung führen (Perle et al. 2014; Simpson und Reid 2014a; Smith et al. 2020; Springer et al. 2020). Dies könnte ein Hinweis darauf sein, dass die angebotenen und absolvierten Trainings- und Schulungsmaßnahmen die Teilnehmenden nicht ausreichend auf die Nutzung der Online-Beratung vorbereiten (Perle et al. 2014). Systematische Evaluationen von Trainings und Schulungen im Bereich der Online-Beratung könnten zukünftig zur weiteren Klärung dieser Hypothese beitragen.

Entsprechend bisheriger Befunde weisen Therapierende auch in der vorliegenden Untersuchung eine negativere Einstellung bezüglich des Online-Settings im Vergleich auf, während interessanterweise Personen aus dem Berufsfeld Coaching den Kernmerkmalen tendenziell und den Rahmenbedingungen signifikant positiver gegenüber eingestellt sind. Die Perspektive aus der Berufsrolle der Beratenden zeigte keine Tendenz. Eine Umfrage von Rabe-Menssen et al. (2020) unter Mitgliedern der Deutschen Psychotherapeuten Vereinigung (DPtV) zeigte, dass 73 % der befragten Therapierenden die Wirksamkeit einer Videobehandlung im Vergleich zu Präsenz-Treffen schlechter einschätzen. Ursächlich für diese Angabe sei die erhöhte Anstrengung und der fehlende direkte Blickkontakt im Rahmen der Therapie. Warum es in der vorliegenden Studie zu den Unterschieden zwischen den beiden Berufsrollen „Coaching“ und „Beratung“ kommt, bleibt jedoch unklar. Zukünftige Forschung in diesem Bereich sollte somit die verschiedenen Berufsperspektiven miterfassen und z. B. um weitere, wie Supervision, ergänzen.

### Einschränkungen

Die durchgeführte Studie besitzt einige Einschränkungen. Eine Einschränkung bildet das neu entwickelte Instrument zur expliziten Erfassung der Einstellung gegenüber Online-Beratung. Die ersten Schritte zur Validierung konnten in der vorliegenden Studie durchgeführt werden, diese muss jedoch in weiteren Untersuchungen fortgeführt werden. Des Weiteren ist die kleine Stichprobe eine weitere Begrenzung, weshalb die Ergebnisse mit Vorsicht interpretiert werden sollten. Es wurden deshalb überall, wo möglich, Effektstärken und standardisierte Regressionsgewichte berichtet. Die mittleren Effektstärken untermauern dabei die Aussagekraft der Ergebnisse.

Zusätzlich fand die Umfrage auf Basis einer Online-Befragung statt. Online-Umfragen können einige systematische Fehler nach sich ziehen, bei denen der Forschende keine Möglichkeit hat, zu intervenieren (Leary 2012). Beispielsweise entstand im vorliegenden Fall womöglich eine selektive Stichprobe, die von vorne herein eine höhere Akzeptanz für Technologien aufweist. Darum sollte bei zukünftigen Studien die Erhebung auch außerhalb des Online-Settings stattfinden.

Weiterhin wurden die Teilnehmenden nicht nach deren subjektiver Bedeutung für die einzelnen Einstellungsmerkmale gefragt, z. B. wie wichtig ihnen die Flexibilität bei Beratungen ist. Zukünftige Studien könnten beispielsweise mithilfe von Multiattributmodellen (Ajzen und Fishbein 1980) die subjektive Bedeutung der Merkmale miterfassen. In ähnlicher Weise wurde verpasst, die Berufserfahrung der Teilnehmenden auf das Online- und das Präsenz-Setting aufzuteilen. So bleibt unklar, ob dies einen Einfluss auf die Ergebnisse hat. Allerdings wurde der Prädiktor „Anzahl bereits durchgeführter Online-Beratungssitzungen“ in beiden Einstellungsdimensionen nicht signifikant, was darauf hindeutet, dass dieser Einfluss zumindest in der vorliegenden Stichprobe zu vernachlässigen ist. Schließlich wurde keine Unterteilung bezüglich der Formen des Online-Settings (Nutzung von video-, telefon- oder chatbasierten Formen) vorgenommen. Die Wahl des Mediums könnte jedoch zu einer stärkeren Differenzierung des Online-Settings beitragen und sollte bei nachfolgenden Untersuchungen berücksichtigt werden.

### Ausblick

Die Digitalisierung der Beratung stellt die Beratenden vor viele neue Herausforderungen. Doch obwohl das Online-Setting nachweislich zum Beratungserfolg führen kann und auch von Seiten der Ratsuchenden weitestgehend akzeptiert wird, sträuben sich noch viele Beratenden gegen das Setting. Die vorliegende Studie untersuchte, welche Aspekte am Online-Setting die Einstellung von Beratenden beeinflusst.

Damit ist diese Studie die erste, die Prädiktoren identifizieren kann, die die Einstellung von Beratenden zur Online-Beratung im direkten Vergleich zur Präsenz-Beratung positiv oder negativ beeinflussen. Die Studie liefert damit nicht nur wertvolle Hinweise für Forschung und Praxis im Bereich der Online-Beratung. Auch Organisationen, Weiterbildungsinstitute und Anwendungsentwicklende können von diesen Erkenntnissen profitieren und eine wesentliche Rolle bei der Veränderung der Einstellung der Beratenden einnehmen. Beispielsweise könnten Aus- und Weiterbildungen auf Grundlage dieser Ergebnisse spezifiziert werden, indem zuvor die Einstellung der Teilnehmenden eingeschätzt und davon individuelle Bedürfnisse abgeleitet werden. Demnach könnte exemplarisch für berufserfahrene Teilnehmende eine andere Schulung organisiert werden, als für unerfahrene Teilnehmende, um dem Ziel näher zu kommen, Vorbehalte gegenüber dem nachweislich wirksamen Online-Setting weiter aufzulösen.

## Supplementary Information






