# Supplementary material for: Attitudes Towards Online-Coaching: A Survey among Counselors, Coaches and Therapists
Source: Coaching Theor. Prax. 2021 Dec 22;7(1):173–84. [Article in German] doi: 10.1365/s40896-021-00061-5 (PMC8693847; doi:10.1365/s40896-021-00061-5)
Supplement: Supplementary file 1 [file 40896_2021_61_MOESM1_ESM.docx]

**Online-Ressourcen 1**

*Items mit Hintergrundinformation zur Itemkonzeption zur Erhebung der Einstellung*

| Items | | Hintergrund | Ursprung |
| --- | --- | --- | --- |
| Dimension 1: Kernmerkmale von Beratungen | | | |
| KK1 | Effektivität | Die Face-to-Face Beratung ist effektiver als die Online-Beratung. | Eells et al. (2014); Eichenberg und Küsel (2016); Rees und Stone (2005); Simpson und Reid (2014 a); Schuster et al. (2018) |
| AK3 | Leichtigkeit, Gefühle zu erkennen/ wahrzunehmen | Die Technologie behindert die Wahrnehmung von audio- und visuellen Reizen. | Rees und Stone (2005); Springer et al. (2020); Wesemann et al. (2010) |
| AK4 | Leichtigkeit, Gefühle zu offenbaren | Die Technologie behindert die Möglichkeit Gefühle zu offenbaren und auszudrücken. | Eells et al. (2014);  Rees und Stone (2005); Simpson und Reid (2014 a); Springer et al. (2020) |
| AK5 | Aufbau einer stabilen Coach-Klient-Beziehung / Therapeut-Patient-Beziehung | Die Technologie behindert die Entwicklung einer therapeutischen Beziehung. | Barrett und Gerskovich (2014); Eells et al. (2014); Rees und Stone (2005); Richards et al. (2016);  Perle et al. (2013);  Simpson und Reid (2014 b);  Simpson et al. (2020); Schuster et al. (2018) |
| KK2 | Gewährleistung eines Schutzraumes (Privatsphäre) | Die Sicherheit meiner Klienten ist nicht gewährleistet. | Smith et al. (2020),  Sammons et al. (2020) |
| AK2 | therapeutischer Nutzen | Der therapeutische Nutzen wird durch die Online-Beratung nicht verbessert. | Barrett und Gerskovich (2014);  Schuster et al. (2018) |
| AK6 | Sinnhaftigkeit der Beratung/Therapie | Face-to-Face Beratung ist sinnvoller. | Simpson & Reid (2014a) |
| AK7 | professionelle Unterstützung durch die Beratung/Therapie | Durch die Beratung über Computer können keine einfühlsamen, emotional ansprechenden, fürsorglichen oder interpersonell kollaborativen Merkmale bereitgestellt werden. | Eells et al. (2014);  Rees und Stone (2005); Simpson und Reid (2014 a); Springer et al. (2020) |
| KO4 | Einsatz von Beratungs-/Therapiemethoden | Die Technologie schränkt mich in meinen Möglichkeiten meinen Patienten zu unterstützen ein. | Rees und Stone (2005); Richards et al. (2018);  Smith et al. (2020) |
| Dimension 2: Rahmenbedingungen von Beratungen | | | |
| KK3 | Durchführbarkeit | Die Online-Beratung ist störungsanfälliger. | Simpson et al. (2020); Springer et al. (2020) |
| KK4 | Flexibilität | Die Technologie schränkt mich in meinen Möglichkeiten meinen Patienten zu unterstützen ein. | Rees und Stone (2005); Richards et al. (2018);  Smith et al. (2020) |
| KK5 | Anonymität | Durch die Online-Beratungen gehen wesentliche Informationen über die zu behandelnde Person verloren. | Smith et al. (2020) |
| KK6 | Vertraulichkeit | Die Online-Beratung ist unsicherer. | Smith et al. (2020) |
| KO1 | Ihren eigenen Arbeitsaufwand als Berater*in/Therapeut*in | Online- Beratung ist arbeitsaufwändiger. | Smith et al. (2020) |
| KO2 | Möglichkeiten zur Intervention in Krisensituationen des Klienten | In Problem- oder Krisensituationen kann ich meinem Klienten nicht helfen. | Rees und Stone (2005); Richards et al. (2018);  Smith et al. (2020) |
| KO3 | Ihren eigenen finanzieller Aufwand als Berater*in/Therapeut*in | Der finanzielle Aufwand für die Durchführung der Online-Beratung ist zu hoch. | Barrett und Gerskovich (2014);  Overholser (2013) |
| Wegen Doppelladung ausgeschlossen | | | |
| AK1 | Qualität | Face-to-Face Beratung ist hochwertiger. | Simpson und Reid (2014a), Simpson et al. (2020); Springer et al. (2020) |

*Anmerkungen*. KK = Kognitive Komponente, AK = Affektive Komponente, KO = Konative Komponente.
