# Supplementary material for: Attitudes Towards Online-Coaching: A Survey among Counselors, Coaches and Therapists
Source: Coaching Theor. Prax. 2021 Dec 22;7(1):173–84. [Article in German] doi: 10.1365/s40896-021-00061-5 (PMC8693847; doi:10.1365/s40896-021-00061-5)
Supplement: Supplementary file 2 [file 40896_2021_61_MOESM2_ESM.docx]

**Online-Ressourcen 2**

*Items mit Hintergrundinformation zur Skalenkonzeption zur Erhebung der Datenschutzwichtigkeit*

| Items | | Hintergrund | Ursprung |
| --- | --- | --- | --- |
| 1 | Die verschlüsselte Datenübertragung im Internet ist mir sehr wichtig. | Sensible Daten sollten unbedingt geschützt und gesichert werden | Kupfer und Mayer (2019);  Rochlen (2004) |
| 2 | Es ist mir wichtig, dass die Tools und Plattformen, die ich bei der Online-Beratung/Therapie nutze, DSGVO sind. | Berufliche Vorschrift, dass die Tools DSGVO konform sind. Angst der Beratenden diese Richtlinie nicht erfüllen zu können. | Hörmann et al. (2019);  Anthony und Goss (2009) |
| 3 | Es ist mir wichtig, dass die Plattform, die ich für die Online-Beratung/Therapie nutze, einen Ansprechpartner für datenschutzrechtliche Fragen hat. | Ein Ansprechpartner kann die Angst und das Unwohlsein verringern und Bedenken hinsichtlich des Datenschutzes verringern. | Hörmann et al. (2019) |
