# Supplementary material for: Attitudes Towards Online-Coaching: A Survey among Counselors, Coaches and Therapists
Source: Coaching Theor. Prax. 2021 Dec 22;7(1):173–84. [Article in German] doi: 10.1365/s40896-021-00061-5 (PMC8693847; doi:10.1365/s40896-021-00061-5)
Supplement: Supplementary file 3 [file 40896_2021_61_MOESM3_ESM.docx]

**Online-Ressourcen 3**

*Übersicht der übernommenen Fragen zur Erfassung der Technikbereitschaft*

| Item | | Erfassung von |
| --- | --- | --- |
| Kurzskala Neyer et al. (2016) | | |
| 1 | Hinsichtlich technischer Neuentwicklungen bin ich sehr neugierig. | TAkz. |
| 2 | Ich finde schnell Gefallen an technischen Neuentwicklungen. | TAkz. |
| 3 | Ich bin stets daran interessiert, die neuesten technischen Geräte zu verwenden. | TAkz. |
| 4 | Für mich stellt der Umgang mit technischen Neuerungen zumeist eine Überforderung dar. | TKomp. |
| 5 | Ich habe Angst, technische Neuentwicklungen eher kaputt zu machen, als dass ich sie richtig benutze. | TKomp. |
| 6 | Den Umgang mit neuer Technik finde ich schwierig, ich kann das meistens einfach nicht. | TKomp. |
| 7 | Ob ich erfolgreich in der Anwendung moderner Technik bin, hängt im Wesentlichen von mir ab. | TKon. |
| 8 | Es liegt in meiner Hand, ob mir die Nutzung technischer Neuentwicklungen gelingt, mit Zufall oder Glück hat das wenig zu tun. | TKon. |
| 9 | Wenn ich im Umgang mit Technik Schwierigkeiten habe, hängt es schlussendlich allein von mir ab, dass ich sie löse. | TKon. |
| Zusätzliche Items aus dem TA-EG von Karrer et al. (2009) | | |
| 10 | Es macht mir Spaß, ein elektronisches Gerät auszuprobieren. | TAkz |
| 11 | Ich kenne die meisten Funktionen der elektronischen Geräte, die ich besitze. | TKomp. |
| 12 | Es fällt mir leicht, die Bedienung eines elektronischen Geräts zu lernen. | TKomp. |
| 13 | Ich kenne mich im Bereich elektronischer Geräte aus. | TKomp. |

*Anmerkungen.* TAkz. = Technikakzeptanz, TKomp. = Technikkompetenzüberzeugungen, TKon. = Technikkontrollüberzeugung.
